# *Myb* expression is critical for myeloid leukemia development induced by *Setbp1* activation

**DOI:** 10.18632/oncotarget.13383

**Published:** 2016-11-16

**Authors:** Nhu Nguyen, Bandana A. Vishwakarma, Kevin Oakley, Yufen Han, Bartlomiej Przychodzen, Jaroslaw P. Maciejewski, Yang Du

**Affiliations:** ^1^ Department of Pediatrics, Uniformed Services University of the Health Sciences, Bethesda, MD, USA; ^2^ Department of Translational Hematology and Oncology Research, Taussig Cancer Institute, Cleveland Clinic, Cleveland, OH, USA

**Keywords:** Setbp1, Myb, myeloid leukemia

## Abstract

*SETBP1* missense mutations have been frequently identified in multiple myeloid neoplasms; however, their oncogenic potential remains unclear. Here we show that expression of *Setbp1* mutants carrying two such mutations in mouse bone marrow progenitors efficiently induced development of acute myeloid leukemias (AMLs) in irradiated recipient mice with significantly shorter latencies and greater penetrance than expression of wild-type *Setbp1*, suggesting that these mutations are highly oncogenic. The increased oncogenicity of *Setbp1* missense mutants could be due in part to their capability to drive significantly higher target gene transcription. We further identify *Myb* as a critical mediator of *Setbp1*-induced self-renewal as its knockdown caused efficient differentiation of myeloid progenitors immortalized by wild-type *Setbp1* and *Setbp1* missense mutants. Interestingly, *Myb* is also a direct transcriptional target of Setbp1 and Setbp1 missense mutants as they directly bind to the *Myb* locus in immortalized cells and dramatically activate a critical enhancer/promoter region of *Myb* in luciferase reporter assays. Furthermore, *Myb* knockdown in *Setbp1* and *Setbp1* missense mutations-induced AML cells also efficiently induced their differentiation in culture and significantly prolonged the survival of their secondary recipient mice, suggesting that targeting MYB pathway could be a promising strategy for treating human myeloid neoplasms with *SETBP1* activation.

## INTRODUCTION

Missense mutations of *SETBP1* are highly recurrent in multiple myeloid neoplasms, including atypical chronic myeloid leukemia [1], chronic myelomonocytic leukemia (CMML) [2], secondary acute myeloid leukemia (sAML) [2], juvenile myelomonocytic leukemia (JMML) [3], and chronic neutrophilic leukemia (CNL) [4, 5], suggesting that they may play an important role in the development of these malignancies. The association of *SETBP1* missense mutations with poor prognosis in many of these diseases further suggests that improved therapeutic strategies for patients with such mutations are critically needed. It remains unclear, however, how *SETBP1* mutations may contribute to the development and progression of these myeloid neoplasms. It has been suggested that these mutations may increase stability of SETBP1 protein [1]. Consistent with this idea, oncogenic activities previously identified with overexpression of wild-type *SETBP1* also have been found to associate with *SETBP1* mutations: inhibition of tumor suppressor protein phosphatase type 2A (PP2A) [6], and transcriptional activation of *Hoxa9* and *Hoxa10* [7]. Recently, we found that wild-type Setbp1 also can function as a transcriptional repressor in suppressing the transcription of tumor suppressor gene *Runx1* in myeloid progenitors [8]. We also showed that overexpression of wild-type *Setbp1* is capable of inducing AML development in a mouse bone marrow transduction and transplantation model [8]. Since the occurrence of *SETBP1* mutations has been associated with disease progression in myeloid neoplasms [2, 3], the leukemogenic capability of wild-type *Setbp1* suggest that these mutations could be responsible for driving leukemic transformation of these diseases. However, the oncogenic potential of *SETBP1* missense mutations *in vivo* remains to be thoroughly examined.

Myb is a helix–turn–helix transcription factor essential for the establishment of definitive hematopoiesis [9]. In adult hematopoiesis, *Myb* also is critical for both T and B cell development [10–12]. The involvement of *Myb* in leukemia development was implicated initially by its involvement in retrovirus-induced transformation of hematopoietic cells [13, 14]. In myeloid leukemias, *MYB* has been shown to be a critical target of known oncogenes, including *HOXA9* and *MLL* fusions [15, 16], and has been identified as a part of a leukemia stem cell maintenance signature [17]. The ability of *Myb* to contribute to leukemogenesis is likely through its direct activation of a number of leukemia-promoting genes including *Myc* [18], *CCNB1* [19], *Bcl2* [18, 20], *Smyd2* [16], and *GFI1* [21], as well as its direct repression of key differentiation regulators containing *Sfpi1*, *Runx1*, *Junb*, and *Cebpb* [22].

Here, we first examine the oncogenic potential of *SETBP1* missense mutations by performing mouse bone marrow transduction and transplantation experiments using *Setbp1* mutants carrying two different recurrent mutations, I871T and D868N. Our study suggests that *SETBP1* mutations possess significantly higher oncogenic potential than wild-type *SETBP1*, which is likely due in part to their capability to drive higher levels of target gene transcription. We also identified *Myb* as a critical and direct target of both wild-type *Setbp1* and *Setbp1* missense mutants.

## RESULTS

### *Setbp1* missense mutations efficiently induce AML development in mice

**Figure 1:**
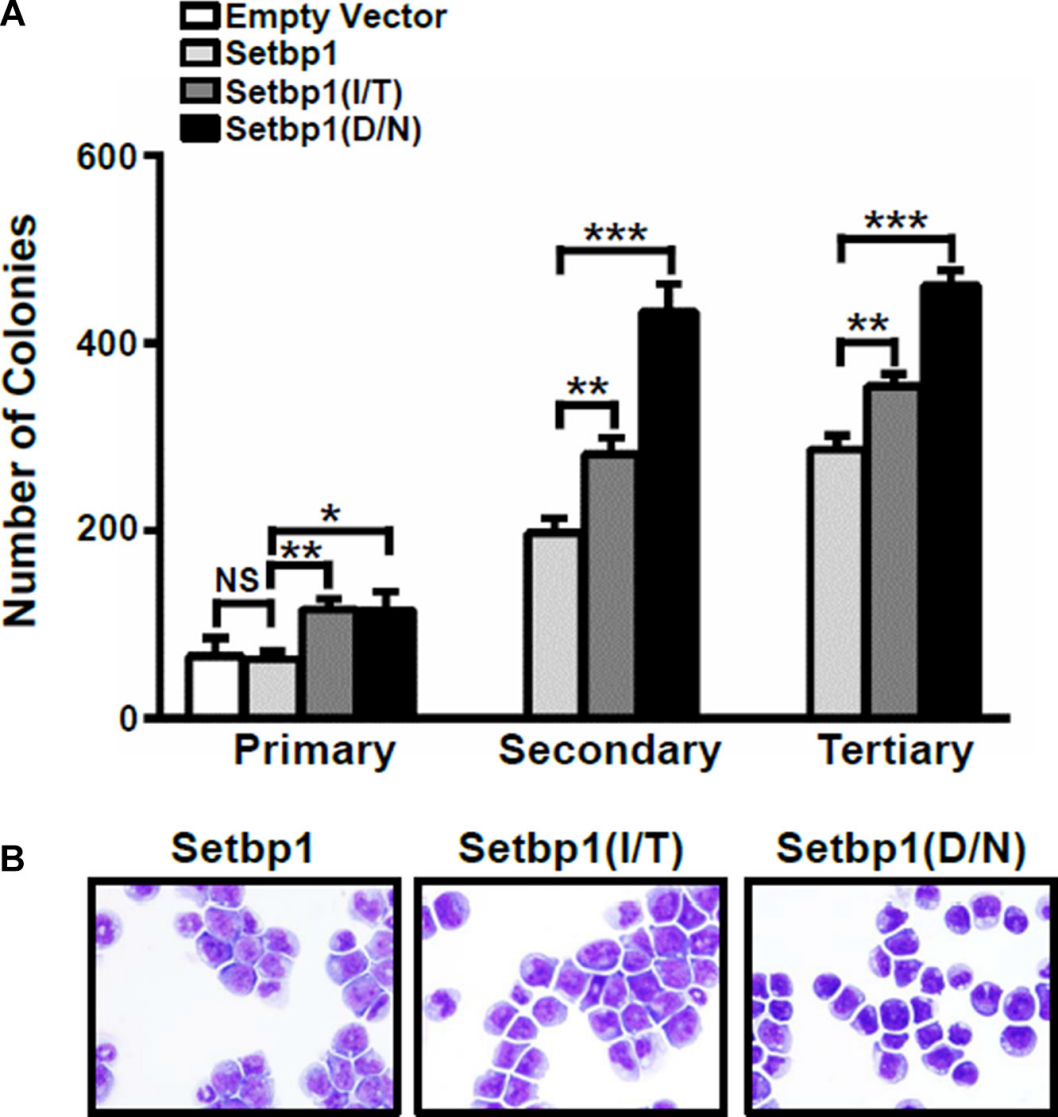
*Setbp1* missense mutants confer more efficient immortalization to bone marrow progenitors compared to wild-type *Setbp1* (**A**) Mean and SD of colony numbers formed by 5 × 10^3^ 5-fluorouracil (5-FU) treated bone marrow (BM) progenitors from C57BL/6 mice transduced with *pMYs-IRES-GFP*, *pMYs-Setbp1-IRES-GFP*, *pMYs-Setbp1(I/T)-IRES-GFP*, or *pMYs-Setbp1(D/N)-IRES-GFP* retrovirus. Infected cells were sorted by GFP expression and plated on methylcellulose medium in the presence of stem cell factor (SCF) (100 ng/ml), interleukin (IL)-6 (10 ng/ml), and IL-3 (6 ng/ml). Cells were re-plated every 5–7 days. (**B**) Wright-Giemsa staining of cells immortalized by transduction with retroviruses expressing the indicated wild-type or mutant *Setbp1* after passaging in liquid media containing SCF and IL-3 for 2.5 months. *P < 0.05; **P < 0.01; ***P < 0.001 (two-tailed Student’s *t* test).

**Figure 2:**
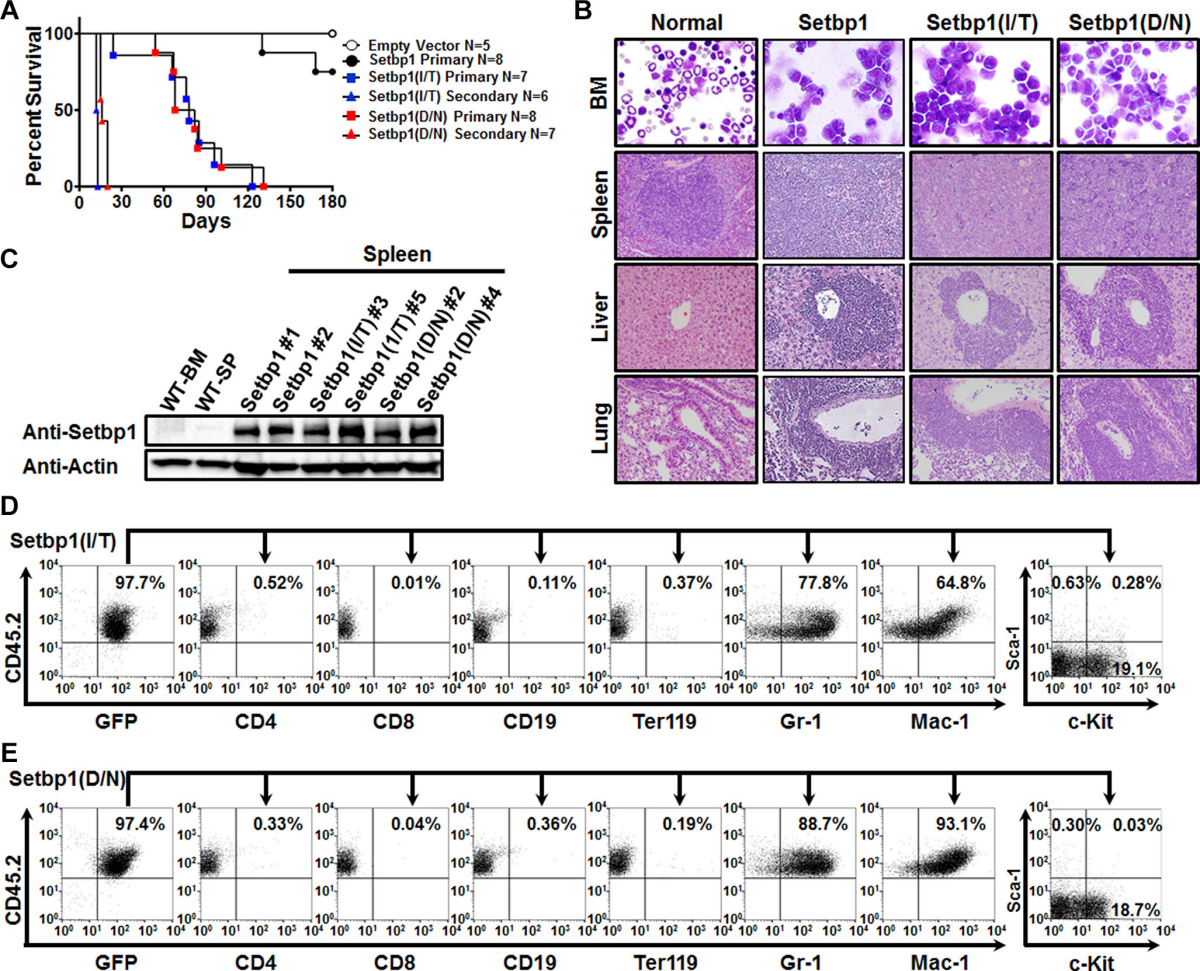
*Setbp1* missense mutations induces AML development (**A**) Survival curves of lethally-irradiated C57BL6-*Ly5.2* mice receiving 5-FU treated bone marrow progenitors transduced with *pMYs-IRES-GFP*, *pMYs-Setbp1-IRES-GFP*, *pMYs-Setbp1(I/T)-IRES-GFP*, or *pMYs-Setbp1(D/N)-IRES-GFP* virus, or 1 × 10^6^ spleen cells from primary leukemic mice. (**B**) Representative cytospin analysis of bone marrow (BM) cells from leukemic mice and H&E staining of spleen, liver, and lung tissue sections showing leukemic infiltration in *Setbp1* mutants-induced leukemic mice. (**C**) Western blotting analysis of Setbp1 and β-Actin in wild-type bone marrow (WT-BM) and spleen (WT-SP), and leukemic spleens induced by wild-type or mutant *Setbp1*. *P < 0.05; **P < 0.01; ***P < 0.001 (two-tailed Student’s *t* test). (**D**) Representative FACS analysis of GFP and CD45.2 double positive leukemia cells from the bone marrow of *Setbp1(I/T)*-induced leukemic mice using the indicated antibodies. Numbers represent the percentages of gated events. (**E**) Representative FACS analysis of GFP and CD45.2 double positive leukemia cells from the bone marrow of *Setbp1(D/N)*-induced leukemic mice using the indicated antibodies. Numbers represent the percentages of gated events.

The association of *SETBP1* missense mutations with disease progression in myeloid neoplasms suggests that the mutations could be responsible for driving leukemic transformation in these diseases. The oncogenic potential of such mutations, however, remains unclear. We previously showed that expression of these mutations in committed myeloid progenitors efficiently induced their immortalization in culture [2]. To further examine the transforming capabilities of these mutations in comparison to wild-type *Setbp1*, we transduced 5-fluorouracil (5-FU) treated bone marrow progenitors from C57BL/6 mice, which are enriched for hematopoietic stem and early progenitor cells, with pMYs retroviruses expressing *Setbp1* mutants with two recurrent mutations I871T and D868N (*pMYs-Setbp1(I/T)-IRES-GFP* and *pMYs-Setbp1(D/N)-IRES-GFP*) or wild-type *Setbp1 (pMYs-Setbp1-IRES-GFP)*, or control empty pMYs virus (*pMYs-IRES-GFP*), and subsequently carried out serial re-plating assays using the purified transduced cells in the presence of SCF, IL-6 and IL-3. Similar transduction efficiencies of 15–20% were detected for all 4 groups when transduced cells were purified by FACS based on GFP expression. As expected, cells infected with empty pMYs virus lost their colony-forming potential after the first plating. In contrast, cells infected with *Setbp1* viruses continued to form large number of colonies with similar size and morphology, even at the third plating, with significantly more colonies formed by cells expressing *Setbp1* mutants than cells expressing wild-type *Setbp1* (Figure 1A). While containing slightly higher levels of Setbp1 protein, mutant Setbp1 colonies expressed significantly lower levels of *Setbp1* mRNA than wild-type Setbp1 colonies (Supplementary Figure S1), suggesting increased protein stability for Setbp1 mutants as reported previously [1, 2]. Consistent with our previous studies using more differentiated myeloid progenitors [2], the cells of these tertiary colonies were immortalized as 10 liquid cultures established from 10 randomly picked colonies of each transduction can be passaged continuously for over a month in the presence of SCF and IL-3 (Figure 1B and data not shown). These immortalized cells display similar surface marker expression profile to cells immortalized previously from committed myeloid progenitors (Supplementary Figure S2) [2]. These cells are also dependent on IL-3 for growth, with faster proliferation observed for cells immortalized by *Setbp1* mutants than cells by wild-type *Setbp1* (Supplementary Figure S3). In order to test the oncogenicity of these mutations *in vivo*, we transplanted freshly transduced cells into lethally-irradiated congenic B6-Ly5.2 recipient mice with supporting normal bone marrow cells. Similar transduction efficiencies of 25–32% were achieved for all three different Setbp1 groups based on GFP positivity before transplantation. The entire cohorts of recipient mice receiving *Setbp1* mutants transduced cells fell ill and had to be euthanized within 131 days after transplantation (Figure 2A). Consistent with our previous studies [8], two of the mice receiving wild-type *Setbp1* transduced cells developed myeloid leukemia in 5 months and all mice receiving empty virus infected cells remained healthy during the same period (Figure 2A). The moribund animals from *Setbp1* mutant groups exhibited enlarged spleens and livers. Closer examination of cells from the spleen and bone marrow of these animals by cytospin analysis revealed a high prevalence of immature myeloid blast cells (Figure 2B), representing 65 ± 5.1% for *Setbp1(I/T)* and 48 ± 5.7% for *Setbp1(D/N)* (Mean ± SD) of bone marrow nucleated cells. The myeloid identity of these cells also was confirmed by flow cytometry analyses showing that they were mostly positive for myeloid markers Gr-1 and Mac-1 while negative for markers of other hematopoietic lineages including CD4, CD8, CD19, and Ter119 (Figure 2D and 2E). Similar to leukemia cells induced by wild-type *Setbp1* [8], only a small fraction of these cells are positive for c-kit and Sca-1 (Figure 2D and 2E). Histological examination of the livers and lungs of these animals also showed infiltrations by these abnormal cells, resembling leukemic mice induced by wild-type *Setbp1* (Figure 2B). We have shown previously that myeloid leukemias induced by wild-type *Setbp1* are transplantable [8]. Similarly, these diseases induced by *Setbp1* mutants are also transplantable as irradiated mice receiving 1 × 10^6^ spleen cells from moribund animals developed the same diseases within 21 days (Figure 2A). All these phenotypic characterizations indicate the development of AML in the sick animals of mutant *Setbp1* groups. As expected, high levels of mutant Setbp1 proteins were detected in these leukemias (Figure 2C), confirming their causal role in leukemia induction in these mice. Consistent with the similar transduction efficiencies detected among groups before serial replating assay and transplantation, the immortalized cells and leukemias induced in all groups have similar viral copy numbers (Supplementary Figure S4), suggesting that insertional mutagenesis was unlikely responsible for the differences in colony-forming potential and leukemia latency and penetrance between Setbp1 mutants and wild-type groups. Taken together, these results suggest that *SETBP1* missense mutations are highly oncogenic, with significantly higher transforming potential than wild-type *SETBP1* in inducing AML development.

### *Setbp1* missense mutants induce higher levels of *Hoxa9* and *Hoxa10* expression in primary bone marrow progenitors than wild-type *Setbp1*

**Figure 3:**
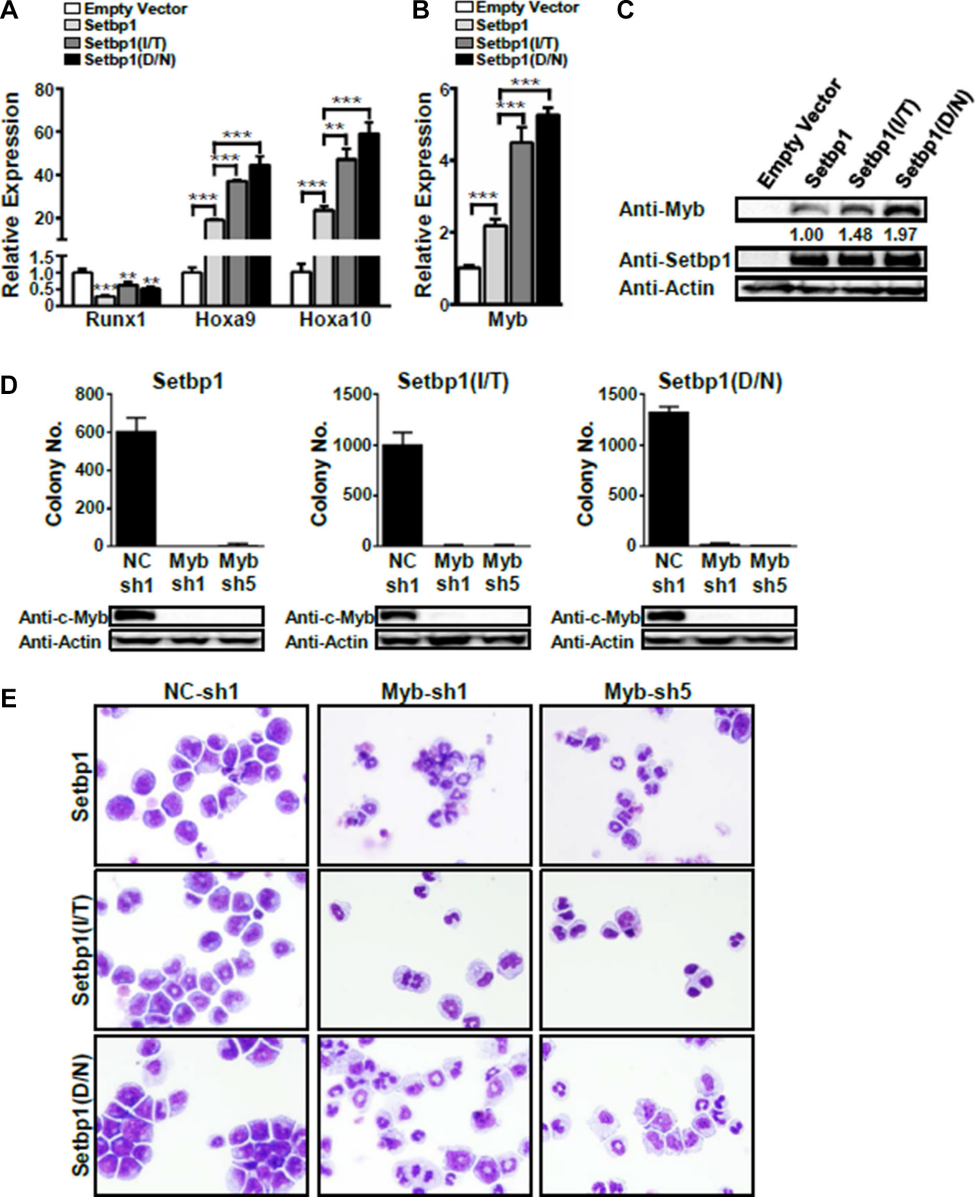
*Myb* activation is essential for the maintenance of *Setbp1* and *Setbp1* missense mutants-induced immortalization Real-time RT-PCR analysis of (**A**) *Runx1, Hoxa9, and Hoxa10* mRNA levels and (**B**) *Myb* mRNA levels in total RNA of primary colonies formed 7 days after infection with the indicated retrovirus. Relative expression levels were calculated by normalizing to *Gapdh* mRNA levels in the same sample and also in cells infected by empty virus. The mean and SD of each relative expression level is shown. (**C**) Representative Western blotting analysis of Myb, Setbp1, and β-Actin protein levels in primary colonies infected as above. The relative Myb/Setbp1 ratios based on densitometry protein quantification are indicated. (**D**) Upper panel, colony forming potential of *Setbp1* and *Setbp1* mutants-immortalized cells plated 48 hrs after infection with lentiviral shRNA targeting *Myb* (Myb-sh1, Myb-sh5) or control shRNA (NC-sh1). Lower panel, Western blotting analysis of Myb protein at 72 hrs after infection in the same cells corresponding to the upper panel. (**E**) Cytospin analyses of *Setbp1* and *Setbp1* mutants-immortalized cells 72 hrs after infection with the indicated lentiviral shRNA. *P < 0.05; **P < 0.01; ***P < 0.001 (two-tailed Student’s *t* test).

We have previously identified *Hoxa9*, *Hoxa10*, and *Runx1* as critical transcriptional targets of wild-type *Setbp1*. It is possible that faster leukemia development induced by *Setbp1(I/T)* and *Setbp1(D/N)* than wild-type *Setbp1* could be due to their capability to induce more potent effects on the transcription of these target genes. Although our previous studies showed that there was no significant difference in *Hoxa9* and *Hoxa10* mRNA levels between cells immortalized by wild-type *Setbp1* and *Setbp1* mutants, this could be resulted from long-term culturing required for the immortalization process. To better address this notion, we examined *Hoxa9*, *Hoxa10*, and *Runx1* mRNA levels in cells of primary colonies freshly formed by purified 5-FU-treated C57BL/6 mouse bone marrow progenitors after transduction by control, *Setbp1*, *Setbp1(I/T)*, or *Setbp1(D/N)* viruses without further culturing. Significantly higher levels of *Hoxa9* and *Hoxa10* mRNA were detected in cells transduced by *Setbp1* mutants than by wild-type *Setbp1*, while no significant difference was observed for *Runx1* mRNA levels between the groups (Figure 3A). Given that less significant differences in the levels of Setbp1 proteins were detected in these cells (Supplementary Figure S1), these results suggest that *Setbp1* missense mutants are more potent transcriptional activators than wild-type Setbp1 and their ability to activate higher levels of target gene transcription could be at least partly responsible for their increased capability to induce leukemia development.

### *Myb* expression is critical for *Setbp1* and *Setbp1* missense mutants-induced immortalization

Since ectopic expression of *Hoxa9* in myeloid progenitors has been shown to activate *Myb* expression, which is known to block differentiation of hematopoietic progenitors, we decided to investigate a potential role of *Myb* in *Setbp1*-induced self-renewal. We assessed first *Myb* expression levels in the same primary colony cells that we used for the analysis of *Hoxa9*, *Hoxa10*, and *Runx1* expression. We found that *Myb* mRNA and protein levels were significantly higher in cells transduced by virus expressing *Setbp1* or *Setbp1* missense mutants than in cells infected by control virus (Figure 3B and 3C). Similar to *Hoxa9* and *Hoxa10* activation, the up-regulation of *Myb* by *Setbp1* missense mutants is considerably greater than by wild-type *Setbp1* (Figure 3B and 3C). In addition, knockdown of wild-type *Setbp1* or *Setbp1* mutants in immortalized cells significantly reduced *Myb* mRNA levels (Supplementary Figure S5). These results suggest that *Myb* expression is up-regulated upon *Setbp1* activation in myeloid progenitors and *Setbp1* missense mutants are more potent activators of *Myb* expression than wild-type *Setbp1*. To test whether *Myb* activation is critical for *Setbp1*-induced immortalization, we knocked down *Myb* in myeloid cells immortalized by *Setbp1*, *Setbp1-I/T*, or *Setbp1-D/N* using two different lentiviral shRNAs. Dramatic reductions in colony formation by the knockdown cells were consistently observed, suggesting that *Myb* expression is critical for the maintenance of these cells (Figure 3D). Functional *Myb* knockdowns by these shRNAs were confirmed by the dramatic reductions in the mRNA levels of known *Myb* transcriptional targets including *Myc* and *Gfi1* (Supplementary Figure S6). Interestingly, cytospin analysis of the knockdown cells also revealed significantly increased neutrophil and macrophage differentiation (Figure 3E), which was confirmed by substantial increases in the expression of myeloid differentiation marker genes including Lyz2 and Cd11b in these cells (Supplementary Figure S7). These results further suggest that *Myb* is critical for the self-renewal of *Setbp1*-immortalized cells (Figure 3E).

### *Myb* is a direct transcriptional target of *Setbp1* and *Setbp1* missense mutations

**Figure 4:**
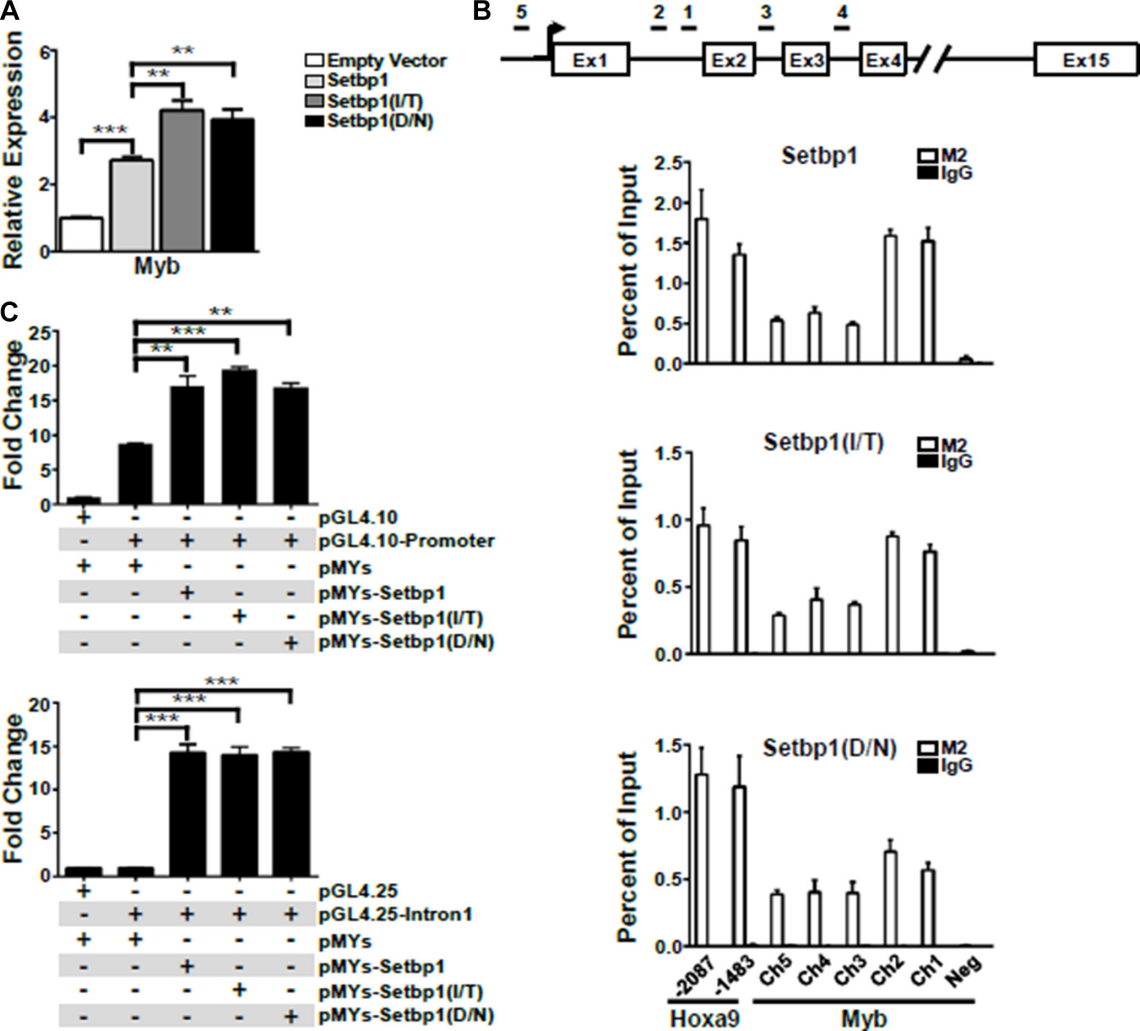
*Myb* is a direct transcriptional target of *Setbp1* and *Setbp1* missense mutants (**A**) Real-time RT-PCR analysis of *Myb* mRNA levels in mouse 5-FU treated BM progenitor cells purified 48 hrs after infection with the indicated retrovirus. Relative expression levels were calculated by normalizing to *Gapdh* mRNA levels in the same sample and also in cells infected by empty virus. The mean and SD of each relative expression level is shown. (**B**) Upper panel, schematic diagram showing locations of genomic regions used for chromatin immunoprecipitation (ChIP) analysis at the *Myb* locus. Lower panels, representative ChIP analyses of indicated regions of *Myb* locus (Ch1–5) and a negative control region (Neg, located at 10,179 bps upstream of *Myb* transcriptional start site) using anti-FLAG M2 antibody in 3xFLAG-tagged *Setbp1* and *Setbp1* mutants-immortalized cells followed by real-time PCR. (**C**) Dual luciferase assays of 293T cells transiently transfected with renilla luciferase reporter plasmid pRL-null, firefly luciferase reporter plasmids including pGL4.10 containing *Myb* immediate promoter (upper panel) and pGL4.25 containing the 1st intron of *Myb* (lower panel), along with the indicated pMYs construct. Mean and SD of ratios between firefly and renilla luciferase activity are shown. *P < 0.05; **P < 0.01; ***P < 0.001 (two-tailed Student’s *t* test).

Ectopic expression of *Setbp1*, *Setbp1(D/N)*, or *Setbp1(I/T)* significantly activates *Myb* transcription in myeloid progenitors 48 hours after transduction (Figure 4A). This rapid induction of *Myb* expression prompted us to further investigate whether *Myb* could be a direct transcriptional target of both wild-type and mutant *Setbp1*. To test this hypothesis, we performed chromatin immunoprecipitation (ChIP) followed by quantitative real-time PCR on myeloid progenitor cells immortalized by 3xFLAG-tagged wild-type or mutant *Setbp1*. We observed significant enrichment for all tested regions of *Myb* locus in chromatin immunoprecipitated with FLAG M2 antibody from all immortalized cell lines examined but not for a control genomic region located over 10 kb upstream of *Myb* locus (Figure 4B). high levels of *Setbp1*-binding comparable to regions of known *Setbp1* target *Hoxa9* were detected at regions within the 1st intron, which has been shown to harbor an alternative promoter for *MYB* [23, 24]. Consistent with the ChIP analyses, *Setbp1* knockdown in these immortalized cells caused significant reduction in *Myb* mRNA levels as early as 36 hours after *Setbp1* knockdown, before any detectable reduction in Hoxa9 protein levels (Supplementary Figure S8), further suggesting that the reduction is not secondary to Hoxa9 reduction. As expected from previous studies [15], Hoxa9 is also involved in the regulation of *Myb* transcription in these cells, as *Hoxa9* knockdown caused significant reduction in *Myb* mRNA levels (Supplementary Figure S9). Therefore, these results suggest that *Setbp1* wild-type and mutants may cooperate with *Hoxa9* to regulate *Myb* transcription. To further test if Setbp1 could directly activate *Myb* transcription, we cloned the –1041 to –1 region of *Myb* immediate promoter and the first intron of *Myb*, which displayed highest levels of Setbp1-binding in our ChIP analyses, upstream of a luciferase reporter and tested effects of ectopic expression of *Setbp1*, *Setbp1(D/N)*, or *Setbp1(I/T)* on luciferase expression in 293T cells. The *Myb* immediate promoter region was tested in the pGL4.10 reporter plasmid without internal promoters. Since the transcriptional start site for the *Myb* alternative promoter in intron 1 has not been mapped, we tested the intron 1 sequence in pGL4.25 which contains a minimal promoter upstream of the luciferase reporter gene. *Setbp1* expression in all cases significantly increased luciferase activity, particularly in the case of *Myb* intron 1 by over 14-fold in comparison to cells transfected with control empty vector (Figure 4C), supporting that wild-type *Setbp1* and *Setbp1* mutants are capable of directly activating *Myb* transcription. Although Setbp1 mutants did not induce higher luciferase activities than wild-type Setbp1 in this assay, it is possible that the higher transcriptional activation activities of Setbp1 mutants in 5-FU treated bone marrow progenitors may require critical hematopoietic specific co-factors which are absent in 293T cells.

### *Myb* is critical for the maintenance of leukemias induced by *Setbp1* and *Setbp1* missense mutations

**Figure 5:**
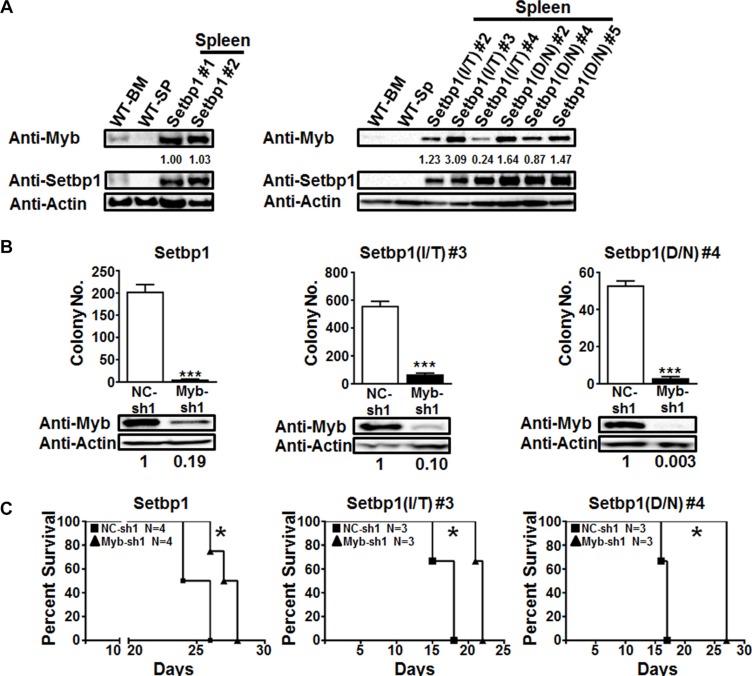
Continuous *Myb* expression is critical for the maintenance of *Setbp1* and *Setbp1* missense mutants-induced AML cells (**A**) Western blotting analysis of Myb and β-Actin in wild-type bone marrow (WT-BM) and spleen (WT-SP), and leukemic spleens induced by *Setbp1* (left panel) and *Setbp1* missense mutants (right panel). (**B**) Mean and SD of colony numbers formed by 1 × 10^4^ *Setbp1*, *Setbp1(I/T)*, or *Setbp1(D/N)* induced leukemic cells 48 hrs after infection with lentiviral shRNA targeting *Myb* (Myb-sh1) or control shRNA (NC-sh1) with the corresponding knockdown efficiency shown at 72 hrs. ***P < 0.001 (two-tailed Student’s *t* test). (**C**) Survival curves of irradiated B6-*Ly5.2* mice transplanted with *Setbp1*, *Setbp1(I/T)* and Setbp1(D/N) induced leukemic cells infected with lentiviral shRNA targeting *Myb* (Myb-sh1) or control shRNA (NC-sh1). Combined results of 2 separate experiments are shown for each survival comparison. *P < 0.05 (Log-rank test).

Consistent with our studies in immortalized cells, high levels of Myb protein were detected in leukemias induced by wild-type *Setbp1* and *Setbp1* missense mutants (Figure 5A). High levels of MYB mRNA were also detected in 2 out of 4 human myeloid neoplasms with SETBP1 missense mutations (Supplementary Figure S10). To extend our observation that *Myb* is a direct and critical target of *Setbp1* and *Setbp1* mutations for inducing immortalization of myeloid progenitors, we assessed whether *Myb* also is required for the maintenance of AML cells induced by their expression. We harvested leukemia cells from secondary recipient mice that have been transplanted with leukemia cells induced by wild-type *Setbp1*, *Setbp1(D/N)*, or *Setbp1(I/T)*, and transduced them immediately with lentivirus containing either a *Myb*-specific shRNA or a non-targeting shRNA. Transduced cells were selected with puromycin at 24 hours after infection and subsequently analyzed for differentiation and colony formation *in vitro* and leukemia induction *in vivo* after transplantation into lethally irradiated secondary recipient mice at 48 hours after infection. As expected, examination of puromycin-resistant cells at 48 hours after infection showed significant reductions in *Myb* protein levels induced by *Myb* shRNA expression (Figure 5B). In contrast to leukemic cells infected with control shRNA, cells with *Myb* knockdown underwent significant differentiation (Supplementary Figure S12) and displayed dramatically reduced colony-forming capabilities on methylcellulose (Figure 5B). These results are consistent with the effects of *Myb* knockdown in *Setbp1*-immortalized cells and suggest that *Myb* may also be important for the self-renewal of AML cells induced by *Setbp1* and its missense mutants. In line with these *in vitro* studies, we also observed significant delays in the onset of AML development in mice transplanted with *Myb* knockdown cells compared to their control counterparts (Figure 5C), suggesting that *Myb* expression is also likely critical for the maintenance of *Setbp1* activation-induced AMLs *in vivo*.

## DISCUSSION

*SETBP1* missense mutations have been identified frequently during disease progression in various myeloid neoplasms; however, it is unclear whether these mutations are potent oncogenic driver mutations for the leukemic transformation of these diseases. We showed previously that expression of these mutations in committed myeloid progenitors efficiently induced their immortalization in culture; however, these cells failed to induce leukemia development when transplanted into lethally-irradiated recipient mice (data not shown). Here we showed that these mutations are highly oncogenic when expressed in hematopoietic stem and early progenitors, as they induced AML development in 100% of the recipient mice within five months. These leukemias are phenotypically similar to leukemias induced by wild-type *Setbp1*. The significantly shorter latency and greater penetrance of this leukemia development compared to that of wild-type *Setbp1*-induced disease induction suggest that *SETBP1* missense mutants are more potent oncogenes. The mechanism(s) underlying this dramatically increased transforming capability of *SETBP1* mutations remains unclear. We found, however, that *Setbp1* mutants induced significantly higher levels of *Hoxa9*, *Hoxa10*, and *Myb* mRNAs than wild-type *Setbp1* in primary hematopoietic progenitor cells. Although the increase in transcription is only about 2-fold in each case, the additive effects of these increases and potentially similar increases in other unknown target gene expression could be very significant and contribute substantially to the dramatically enhanced leukemia development. Since SETBP1 missense mutants have been shown to induce greater PP2A inhibition than wild-type SETBP1, it is possible that increased PP2A inhibition by Setbp1 mutants may also contribute significantly to faster leukemia development in our study. *SETBP1* missense mutations have been shown to increase stability of the protein, which could cause higher levels of target gene transcription. However, our analysis of colonies formed by *Setbp1*-expressing cells showed that significantly higher target gene mRNA levels occurred with comparable levels of Setbp1 proteins present in these cells, suggesting that mechanism(s) other than *Setbp1* protein stability may be responsible for this effect. It is possible that Setbp1 mutants have enhanced DNA-binding activity and/or the interaction(s) of Setbp1 with unknown key transcriptional co-factors or repressors may be affected by the mutations. During the preparation of this manuscript, in contrast to the potent transforming capability of *Setbp1* missense mutants observed in our study, one study reported that overexpression of mutant *SETBP1* with the D868N mutation was not capable of inducing leukemia in a mouse bone marrow transduction and transplantation model similar to the system used here [25]. Different from our study which used untagged Setbp1 mutant proteins, a c-terminally tagged SETBP1 mutant protein was examined in this study. Therefore, although the reason for this discrepancy is currently unclear, it is possible that the addition of a tag to the c-terminus of SETBP1 protein may interfere with its transforming activity.

We also identified *Myb* as another critical direct target of *Setbp1* in conferring self-renewal capability to myeloid progenitors. Consistent with the previously identified role of *Myb* in blocking differentiation, *Myb* knockdowns led to differentiation in myeloid progenitors immortalized by both wild-type and mutant *Setbp1*. Interestingly, we also found that *Myb* is a direct transcriptional target of *Setbp1* by ChIP analysis. Bindings by wild-type or mutant Setbp1 were detected not only in *Myb* promoter regions but also in *Myb* intron 2 and 3, further suggesting that Setbp1 may regulate both transcriptional activation and elongation at the locus. Given that *Myb* has been shown to be a target of *Hoxa9*, and as expected *Hoxa9* knockdown in *Setbp1*-immortalized cells led to significant decrease in *Myb* mRNA levels, a possible cooperation may exist between Setbp1 and Hoxa9 in transcriptional regulation of *Myb*. Although we did not detect any positive effects of *Hoxa9* expression on transcription from the *Myb* promoter or intron 1 in luciferase assays when it was tested alone and in combination with expression of wild-type or mutant *Setbp1* (data not shown), this could be due to the absence of some critical myeloid specific transcriptional co-factor(s) for Hoxa9 in 293T kidney epithelial cells. The nature of this cooperation merits further investigation in the future.

Identification of *Myb* as a critical target of wild-type and mutant *Setbp1* in our study suggest that inhibition of *MYB* could be a potential strategy for treating myeloid neoplasms with *SETBP1* activation. *Myb* knockdown in mouse leukemias induced by wild-type and mutant *Setbp1* was not able to induce dramatic extension of recipient survival in our study. However, we detected significant levels of Myb protein in the leukemia cells developed from the knockdown groups, suggesting that the leukemia development in these mice might be due to expansion of leukemia cells that had escaped knockdown (Supplementary Figure S13). Consistent with our mouse studies, we detected significantly higher *MYB* mRNA levels in one secondary AML and one RAEB-2 patient samples with *SETBP1* missense mutations than in healthy bone marrow samples, which normally express significant levels of *MYB* due to its expression in normal myeloid progenitors and also in erythroid and lymphoid cells [26] (Supplementary Figure S8). Although high levels of *MYB* expression were not observed in two CMML and RAEB-1 samples that harbor *SETBP1* missense mutations, it is possible that the up-regulation of *MYB* transcription by *SETBP1* is specific to immature cells and the lack of *MYB* overexpression in these samples is due to their lower blast counts. In supporting the critical requirement for *MYB* in *SETBP1* mutant cases without high levels of *MYB* expression, *MYB* knockdown in primary cells from the CMML patient dramatically inhibited their colony-forming capability (Supplementary Figure S11). Leukemia cells have been shown to be more sensitive to reduction of MYB activity than normal hematopoietic progenitors [27, 28]. In support of these findings, it was reported recently that interaction of Myb with p300 is required for *Myb*-mediated leukemia transformation, but is less critical for normal hematopoiesis [29]. Interestingly, the triterpenoid Celastrol has been recently identified as an inhibitor of this interaction and further shown to be effective in inhibiting the growth of mouse AML cells *in vitro* and *in vivo*, while sparing the expansion of normal bone marrow progenitors [30]. Our results here suggest that Celastrol could also be effective for treating *SETBP1* activation-induced neoplasms.

## MATERIALS AND METHODS

### Mice

C57BL/6 and B6-*Ly5.2* female mice (7–12 weeks old; Charles River, Frederick, MD) were maintained in the animal facility of Center for Laboratory of Animal Medicine at Uniformed Services University of the Health Sciences (USUHS, Bethesda, MD). All mouse experiments were carried out according to protocols approved by the USUHS Institutional Animal Care and Use Committee.

### Patient samples

Primary human cells were collected after signing the informed consent, according to the protocols approved by the Institutional Review Board of Cleveland Clinic in accordance with the Declaration of Helsinki.

### Retrovirus generation

The *pMYs-Setbp1-IRES-GFP*, *pMYs-Setbp1(I/T)-IRES-GFP*, and *pMYs-Setbp1(D/N)-IRES-GFP* and the corresponding 3XFLAG-tagged Setbp1 retroviral construct was described previously [2, 7]. High titer retroviruses were produced by transient transfection of Plat-E cells using Fugene-6 (Roche, Indianapolis, IN). Viral titer was assessed by serial dilution and infection of NIH-3T3 cells.

### Retroviral transduction and bone marrow transplantation

5-FU treated bone marrow cells from C57BL/6 mice were extracted and expanded in culture as described [8]. Subsequently, these expanded BM cells were infected two times with high-titer retrovirus carrying *Setbp1* cDNA (*pMYs-Setbp1-IRES-GFP*), *Setbp1(I/T)* cDNA (*pMYs-Setbp1(I/T)-IRES-GFP*), *Setbp1(D/N)* cDNA (*pMYs-Setbp1(D/N)-IRES-GFP*) or *GFP* only (*pMYs-IRES-GFP*) on retronectin-coated plates. For transplantation, 1 × 10^6^ transduced BM cells were injected into the tail vein of each lethally-irradiated (1100 rads from ^137^Cs source) B6-*Ly5.2* female mouse along with 7.5 × 10^5^ supporting bone marrow cells from un-irradiated B6-*Ly5.2* mice. Transplanted mice were aged and closely monitored for signs of leukemia development. For secondary transplantation, 1 × 10^6^ spleen cells from primary recipients with leukemia were injected into lethally irradiated secondary recipients, along with 7.5 × 10^5^ supporting bone marrow cells.

### Lentiviral production, infection, and analysis

pLKO.1 lentiviral constructs containing shRNAs including NC-sh1 and GFP-sh1 were purchased from Sigma Aldrich (NC-sh1, SHC002; GFP-sh1, SHC005; St. Louis, MO). Myb-sh1 and –sh5 were generated by cloning previously described *Myb* targeting sequences [17] into pLKO.1. MYB-sh1 and MYB-sh5 were also cloned in pLKO.1 and their targeting sequences are 5′ GGA AAG TTA TTG CCA ATT ATC 3′ and 5′ GTG GCA GAT GCA CCG AAT ATT 3′ respectively. Infectious lentivirus were generated as described previously [8]. Transduced cells were selected with puromycin (2 μg/ml) at 24 hours after transduction. Colony formation assays were performed at 48 hours after infection using 1 × 10^4^ puromycin-resistant cells on IMDM methylcellulose medium supplemented with 20% horse serum, mouse SCF (100 ng/ml) and IL-3 (10 ng/ml), and puromycin (2 μg/ml). Colony numbers were counted after 7 days.

### Flow cytometry

Flow cytometry analysis of mouse bone marrow and spleen samples was performed using BD LSRII flow cytometer. Leukemic bone marrow and spleen cells were blocked by incubation with anti-FcγR-II/III and subsequently stained with antibodies against markers for myeloid (Gr-1, Mac-1), erythroid (Ter-119), B (CD19) and T (CD4, CD8) lineages and also makers for hematopoietic stem and progenitors (c-kit and Sca-1). Dead cells were excluded by staining with Sytox Blue (Invitrogen, Carlsbad, CA, USA).

### Chromatin immunoprecipitation (ChIP)

Mouse myeloid progenitors immortalized by FLAG-tagged *Setbp1* were generated as described [7]. ChIP analyses were performed using ChIP-IT Express kit (Active Motif, Carlsbad, CA, USA). Immunoprecipitations were performed using FLAG M2 (Sigma-Aldrich,St. Louis, MO, USA) and mouse IgG (G3A1, #5415, Cell Signaling Technologies, Danvers, MA, USA). Chromatin DNA was purified using Active Motif PCR Purification Kit (Active Motif) and quantified by real-time PCR. The ChIP primers used for the Hoxa9 promoter were described previously [31]. Myb locus-specific ChIP primers used in this study include: Myb-Ch1 S, 5′ TCA ATG CAT GCA GCA ATT AGG 3′; Myb-Ch1 AS, 5′ GAG GAG CCC ACC AGG TGT TT 3′; Myb-Ch2 S, 5′ TTC GAC AAA GGA CCC GAA AT 3′; Myb-Ch2 AS, 5′ CAT CTA GCT TGA AAT CAG CCT TTG A 3′;Myb-Ch3 S, 5′ GGG CCT GAG CAG GAC AGA 3′; Myb-Ch3 AS, 5′ TCC TGA GAA CAG GTG GAA GCA 3′; Myb-Ch4 S, 5′ TGG CTG AAG ATG CGT TAG TGA 3′; Myb-Ch4 AS, 5′ GGA GGC ACT TCA GTT CCT TAG G 3′; Myb-Ch5 S, 5′ CAT TTT TCA ATC TCC TCC CAG ATC 3′; Myb-Ch5 AS, 5′ GTG AGG GAG GAA GGG CTT ATG 3′.

### Western blotting analysis

For Western blotting analysis, cells were washed twice with cold PBS and then whole cell lysates were prepared by direct lysis of cell pellets in heated 2X SDS sample buffer. Samples were resolved on 4–12% Tris-Glycine gels (Life Technologies, Carlsbad, CA) followed by transferring onto nitrocellulose membranes (Bio-Rad, Hercules, CA). Primary antibodies used include anti-Setbp1 (16841-1AP, Proteintech, Chicago, IL), anti-Myb (05–175, Millipore, Temecula, CA), anti-Hoxa9 (07–178, Millipore, Temecula, CA), and Anti-β-Actin (ab8224, Abcam, Cambridge, MA). Secondary antibodies used include goat anti-rabbit IgG-HRP (SC-2004, Santa Cruz Biotechnology, Dallas, TX) and anti-mouse IgG-HRP (A-9044, Sigma Aldrich). Protein bands were visualized by incubation with SuperSignal West chemiluminescent substrate (Pierce, Thermo Fisher Scientific, Rockford, IL) and quantified using Image Lab software (Bio-Rad, Hercules, CA).

### Real-time RT-PCR

For real-time RT-PCR, total RNA was extracted from cells using Nucleospin RNA (Clontech, Duren, Germany). Oligo-dT-primed cDNA samples were prepared using Superscript III (Invitrogen), and real-time PCR analysis was performed in triplicates using SYBR green detection reagents (Invitrogen) on a 7500 real time PCR system (Applied Biosystems). Relative changes in expression were calculated according to the ΔΔCt method. The cycling conditions were 50°C for 2 minutes followed by 95°C for 2 minutes, and then 40 cycles of 95°C for 15 seconds and 60°C for 1 minute. The following gene-specific primer sequences were used: Setbp1 S, 5′ CTG CTC ACT GTG GAG ACG ATT C 3′; Setbp1 AS, 5′ TTC TTA TCC AGC ACA CCA AGC TT 3′; Hoxa9 S, 5′ TGT CTC CTC TCC CCC AAA CC 3′; Hoxa9 AS, 5′ GAG ATG AGG CCT GGG ATTTAG A 3′; Hoxa10 S, 5′ CCA GCC CTG GGT AAA CTT AGC 3′; Hoxa10 AS, 5′ CATTGA CCT CAG GCC AGA CA 3′; Runx1 S, 5′ GCA GGC AAC GAT GAA AAC TAC T 3′; Runx1 AS, 5′ GCA ACT TGT GGC GGA TTT GTA 3′;β–Actin S, 5′ CCT CCC TGG AGA AGA GCT A 3′; β–Actin AS, 5′ TCC ATA CCC AAG AAG GAA G 3′; Gapdh S, 5′ AGG TCG GTG TGA ACG GAT TTG 3′; Gapdh AS, 5′ TGTAGACCATGTAGTTGAGGTCA 3′; Myb S, 5′ CCA TGA AAG CTC GGG CTT AG 3′; Myb AS, 5′ CTC GAC ATG GTG TCA GTT GTG 3′; Gfi-1 S, 5′ CCC CGA CTC TCA GCT TAC C 3′; Gfi-1 AS, 5′ GCA CAG TGA CTT CTC CGA CG 3′; Myc S, 5′ ACA GCA GCT CGC CCA AAT C 3′; Myc AS, 5′ AGC AGC GAG TCC GAC GAA 3′; MYB S, 5′ GAA AGC GTC ACT TGG GGA AAA 3′; MYB AS, 5′ TGT TCG ATT CGG GAG ATA ATT GG 3′; GAPDH S, 5′ CAC ATG GCC TCC AAG GAG TAA 3′; GAPDH AS, 5′ TGA GGG TCT CTC TCT TCC TCT TGT 3′.

### Luciferase assay

For construction of luciferase reporter plasmids, *Myb* immediate promoter sequence (–1041 to –1) was amplified by PCR and cloned into pGL4.10 between *Xho*I and *Hind*III sites while *Myb* intron1 sequence (+289 to +4147) was cloned into pGL4.25 with a minimal promoter also using *Xho*I and *Hind*III sites. Transfections were carried out using Fugene 6 (Roche) and dual luciferase activities were measured at 48 hours after transfection using the Dual Luciferase Reporter Assay system (Promega) and a Centro XS3 Microplate Luminometer (Berthold Technologies). Primer sequences for cloning Myb promoter and intron 1 sequences: Myb-Promoter S, 5′ CGC GCT CGA GCT CAT GTG GTG GCC CCA AAC 3′; Myb-Promoter AS, 5′ CGC GAA GCT TCC TCC CGC CAA ATC TGG CGC CCC TGC A 3′; Myb-Intron1-S, 5′ CGC GCT CGA GGT AAT GGG GAG GCT GAG AG 3′; Myb-Intron1-AS, 5′ TGG ACA CAC ATG GGC CAA AG 3′.

### Statistical analysis

Sample sizes and animal numbers were determined by previous experiences. No samples were excluded from analyses. All data were analyzed by two-tailed Student’s *t-test* except that survival curves were compared by Log-rank test. The researchers were not blinded during sample collection and analysis.

## SUPPLEMENTARY FIGURES


