# Supplementary material for: Myb expression is critical for myeloid leukemia development induced by Setbp1 activation
Source: Oncotarget. 2016 Nov 16;7(52):86300–12. doi: 10.18632/oncotarget.13383 (PMC5349915; doi:10.18632/oncotarget.13383)
Supplement: Supplementary file 1 [file oncotarget-07-86300-s001.pdf]

Myb expression is critical for myeloid leukemia development induced by *Setbp1* activation

Supplementary Materials

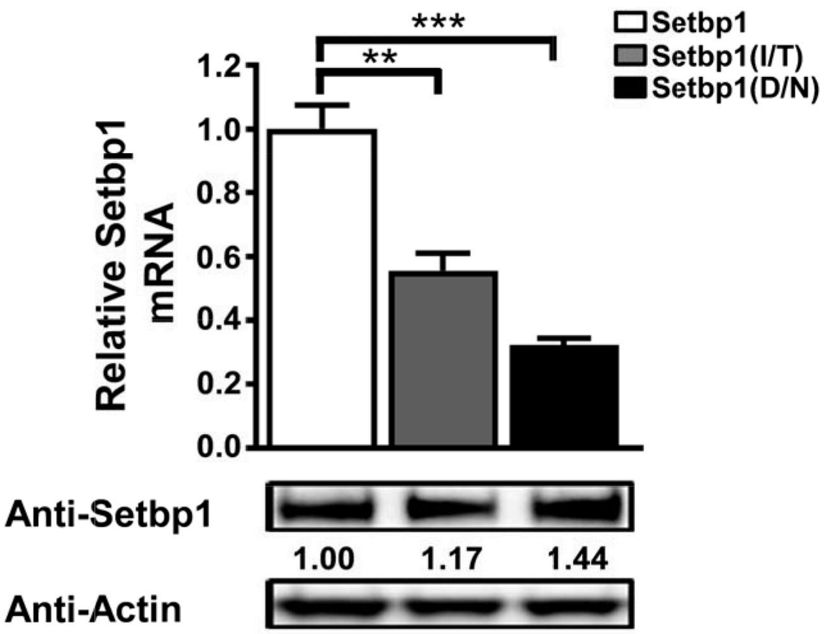

Supplementary Figure S1: *Setbp1* expression in primary colonies formed by hematopoietic stem and progenitor cells transduced by wild-type or mutant *Setbp1* virus. Top panel, real-time RT-PCR analysis of *Setbp1* mRNA levels in total RNA from primary colony cells generated by 5-FU-treated mouse bone marrow progenitors transduced by indicated pMYs viruses expressing wild-type or mutant *Setbp1*. Relative expression levels were calculated by normalizing to β-*Actin* mRNA levels in the same sample and also levels in cells infected by wild-type *Setbp1* virus. The mean and SD of each relative expression level is shown. Lower panels, representative Western blotting analysis of Setbp1 and β-Actin protein levels in the same cells as in the upper panel. Relative levels of Setbp1 protein in each sample after normalizing to β-Actin protein levels are indicated. Protein bands were quantified using Quantity One data analysis software (Bio-Rad). Note that cells infected by wild-type *Setbp1* virus have significantly higher *Setbp1* mRNA levels but lower Setbp1 protein levels than the other two groups, suggesting that wild-type Setbp1 protein is less stable than its missense mutants.

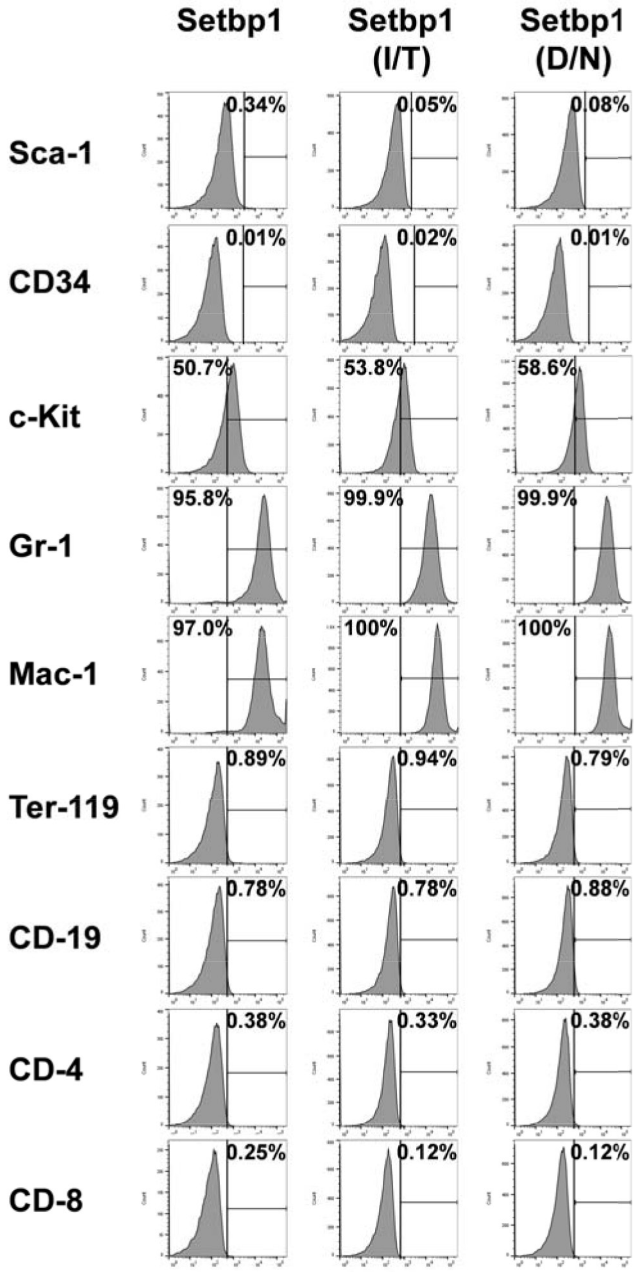

Supplementary Figure S2: Myeloid progenitors immortalized by wild type and mutant *Setbp1* share similar surface marker expression profiles. FACS analysis of indicated marker expression by representative immortalized myeloid progenitors generated by transduction of 5-FU treated mouse bone progenitors with wild-type or mutant *Setbp1* expressing retrovirus. Dead cells were excluded by staining with Sytox Blue (Molecular Probes). All samples were analyzed on a BD LSRII flow cytometer.

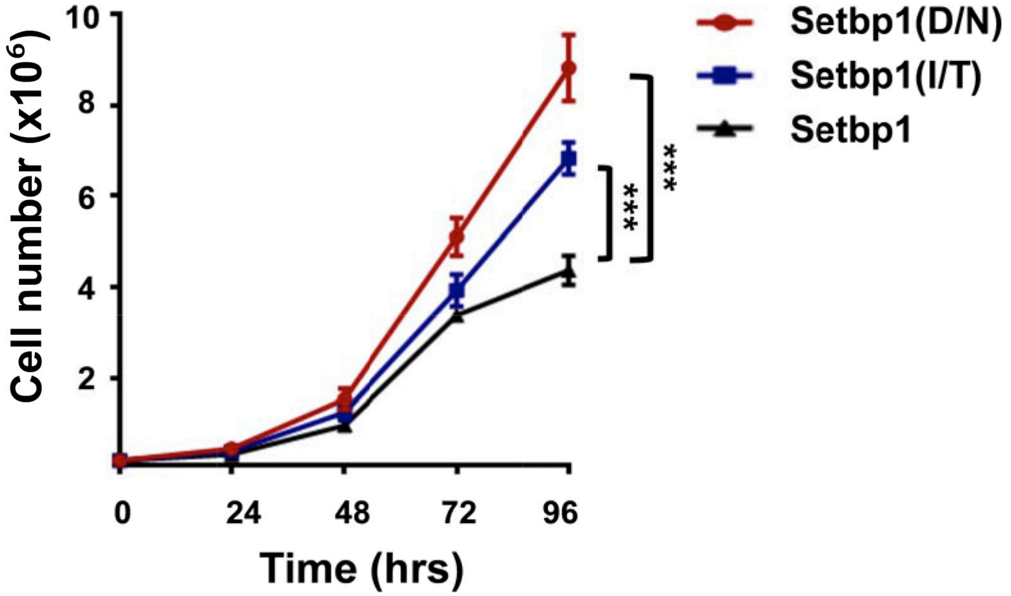

Supplementary Figure S3: Expansion of myeloid progenitors immortalized by wild-type or mutant *Setbp1* in liquid media. Cells were cultured in the presence of SCF and IL-3 over a 96-hour period. Representative results from two independent lines immortalized by each retroviral construct are presented. Cell numbers were counted by trypan blue staining.

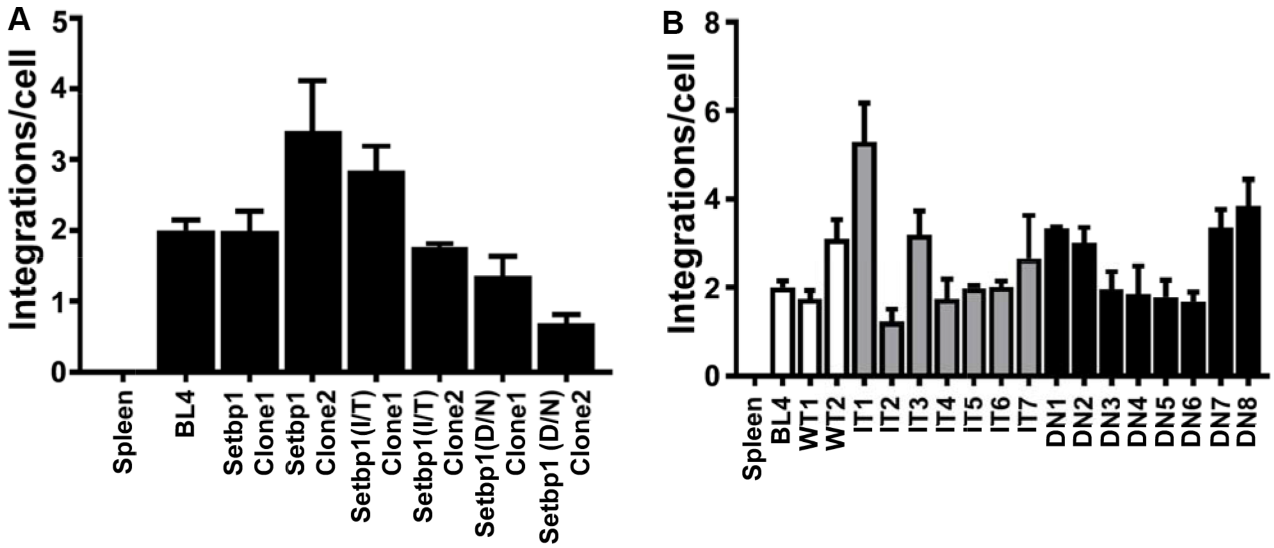

Supplementary Figure S4: Comparable numbers of viral integrations are present in immortalized myeloid progenitors as well as leukemias induced by wild-type *Setbp1* and its missense mutants. (**A**). Viral integrations per cell in 2 randomly selected myeloid progenitor lines immortalized by wild-type *Setbp1*, *Setbp1(I/T)*, or *Setbp1(D/N)* are shown. Viral integrations in the cells were detected by real-time PCR analysis of *GFP* cDNA in their genomic DNA. Integration number per cell for each sample was calculated by normalizing *GFP* levels to endogenous Lipoprotein lipase (Lpl) gene levels in the same sample and also *GFP* levels in BL4 leukemia cells, which contain 2 viral integrations/cell by Southern blotting analysis[1]. As a negative control, genomic DNA from normal C57BL/6 mouse spleen (SP) was also analyzed. (**B**). Viral integrations per cell in leukemic spleens induced by wild-type *Setbp1* (WT1-2), *Setbp1(I/T)* (IT1-7), or *Setbp1(D/N)* (DN1-8) were quantified as described in (A). The mean and SD of each integration number is shown. Primer sequences for detecting *GFP* and *Lpl*: *GFP* S, 5′- AAG CTG ACC CTG AAG TTC ATC TGC -3′; *GFP* AS, 5′- CTT GTA GTT GCC GTC GTC CTT GAA -3′; *Lpl* S, 5′- GGA TGG ACG GTA AGA GTG ATT C -3′; *Lpl* AS, 5′- ATC CAA GGG TAG CAG ACA GGT-3′.

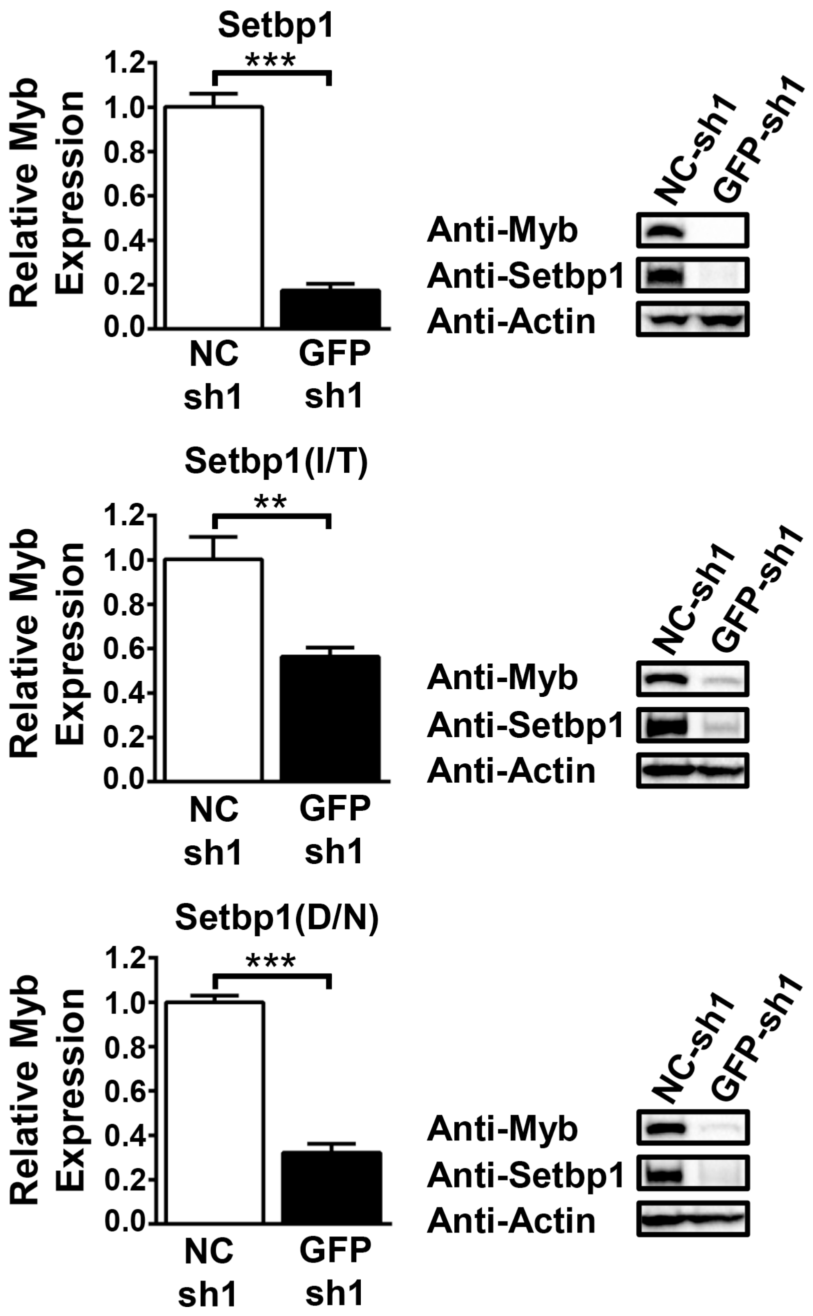

Supplementary Figure S5: Knockdown of *Setbp1* reduces *Myb* mRNA and protein levels. Left panels, real-time RT-PCR analysis of *Myb* mRNA levels using total RNA isolated from *Setbp1* or *Setbp1* mutant immortalized cells infected with a lentiviral shRNA targeting *Setbp1* (GFP-sh1) compared to same cells transduced with control lentiviral shRNA (NC-sh1) at 72 hours after infection. Relative expression levels were calculated by normalizing to β-*Actin* mRNA levels in the same sample and also in cells infected by NC-sh1 virus. The mean and SD of each relative expression level is shown. Right panels, Western blotting analysis of the same cells probed with the indicated antibodies.

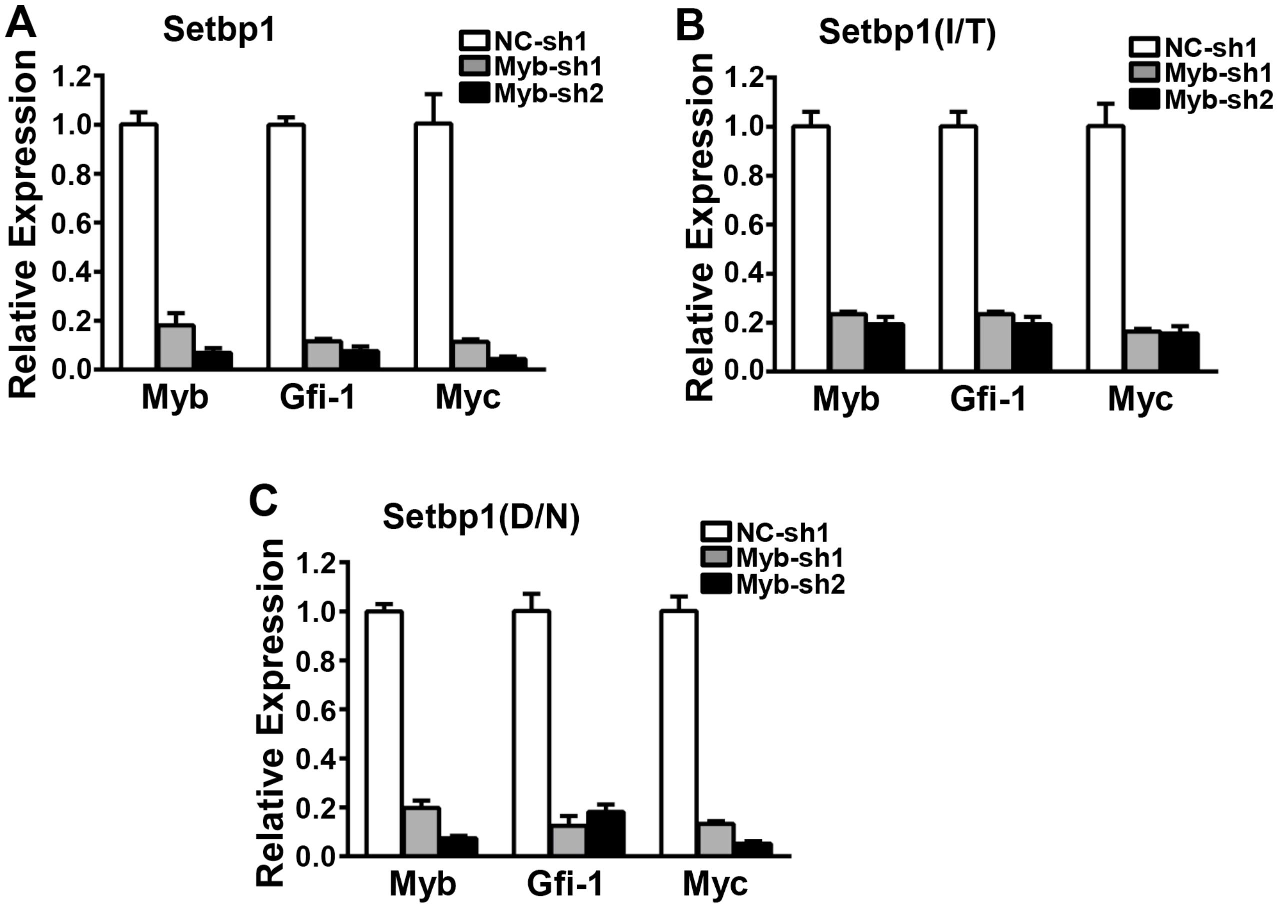

Supplementary Figure S6: Knockdown of *Myb* significantly reduces *Gfi-1* and *Myc* mRNA levels. Real-time RT-PCR analysis of *Myb*, *Gfi-1*, and *Myc* mRNA levels using total RNA isolated from (**A**) *Setbp1*, (**B**) *Setbp1(I/T)*, or (**C**) *Setbp1(D/N)* immortalized cells infected with lentiviral shRNAs targeting *Myb* (Myb-sh1 and Myb-sh2) compared to the same cells infected with control lentiviral shRNA (NC-sh1) at 72 hrs after infection. Relative expression levels were calculated by normalizing to β-*Actin* mRNA levels in the same sample and also in cells infected by NC-sh1 virus. The mean and SD of each relative expression level is shown.

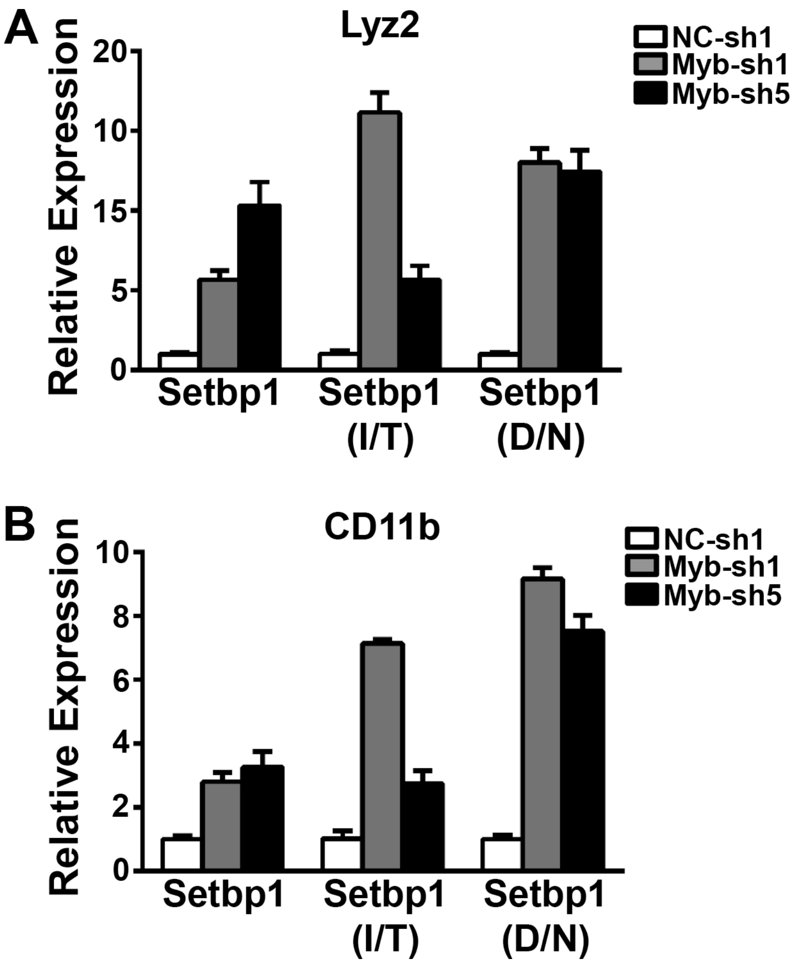

Supplementary Figure S7: Knockdown of *Myb* significantly increases expression of myeloid differentiation marker genes in myeloid progenitors immortalized by wild-type or mutant *Setbp1*. (**A**) Real-time RT-PCR analysis of *Lyz2* mRNA levels at 72 hrs after infection with the indicated *Myb*-specific lentiviral shRNAs (Myb-sh1 and –sh2) and negative control shRNA (NC-sh1) in myeloid progenitors immortalized by *Setbp1*, *Setbp1(I/T)*, or *Setbp1(D/N)*. Relative expression levels were calculated by normalizing to β-*Actin* mRNA levels in the same sample and also in cells infected by NC-sh1 virus. The mean and SD of each relative expression level is shown. (**B**). Real time RT-PCR analysis of *Cd11b* mRNA in the same samples as in (A). Relative expression levels were similarly calculated.

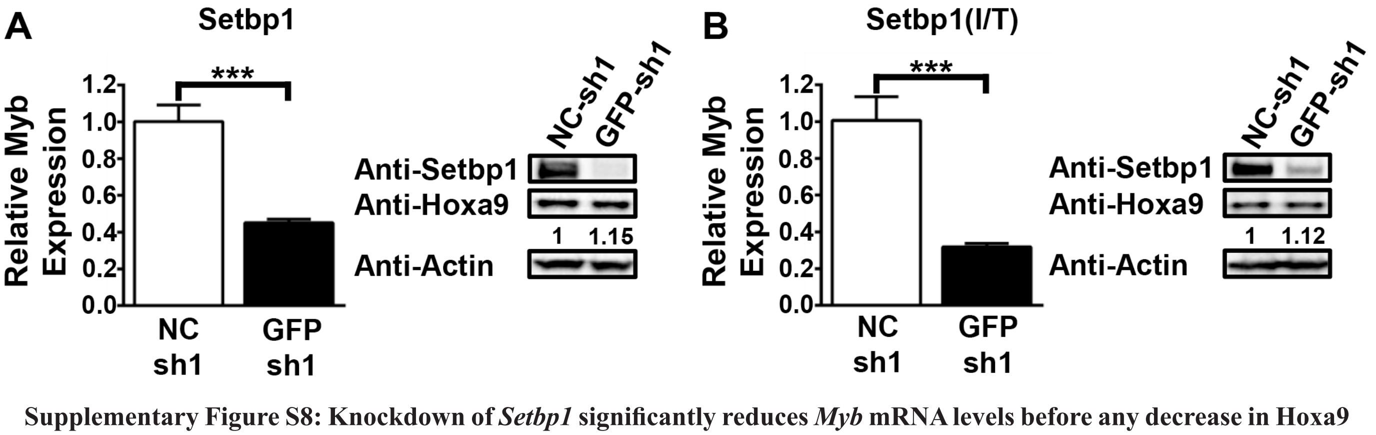

Supplementary Figure S8: Knockdown of *Setbp1* significantly reduces *Myb* mRNA levels before any decrease in Hoxa9 protein levels. Left panels, real-time RT-PCR analysis of *Myb* mRNA levels at 36 hours after infection with the indicated lentiviral shRNAs in (**A**) *Setbp1* and (**B**) *Setbp1(I/T)* immortalized cells. Relative expression levels were calculated by normalizing to β-*Actin* mRNA levels in the same sample and also in cells infected by NC-sh1 virus. The mean and SD of each relative expression level is shown. Right panel, corresponding Western blotting analysis of Setbp1, Hoxa9 and β-Actin proteins in the same cells. Relative Hoxa9 protein levels after normalization to β-Actin are indicated.

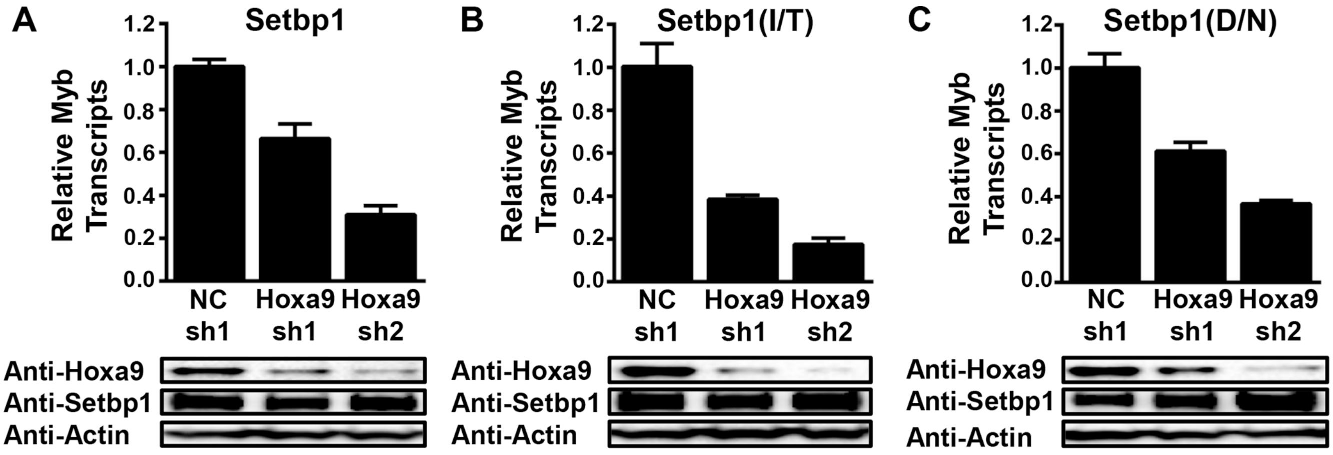

Supplementary Figure S9: Knockdown of *Hoxa9* reduces *Myb* mRNA levels. Upper panels, real-time RT-PCR analysis of *Myb* mRNA levels using total RNA isolated from (**A**) *Setbp1*, (**B**) *Setbp1(I/T)*, or (**C**) *Setbp1(D/N)* immortalized cells infected with lentiviral shRNAs targeting *Hoxa9* (Hoxa9-sh1 and Hoxa9-sh2) [2] compared to same cells transduced with control lentiviral shRNA (NC-sh1) at 72 hrs after infection. Relative expression levels were calculated by normalizing to β-*Actin* mRNA levels in the same sample and also cells infected by NC-sh1 virus. The mean and SD of each relative expression level is shown. Lower panels, Western blotting analyses of the same cells using the indicated antibodies.

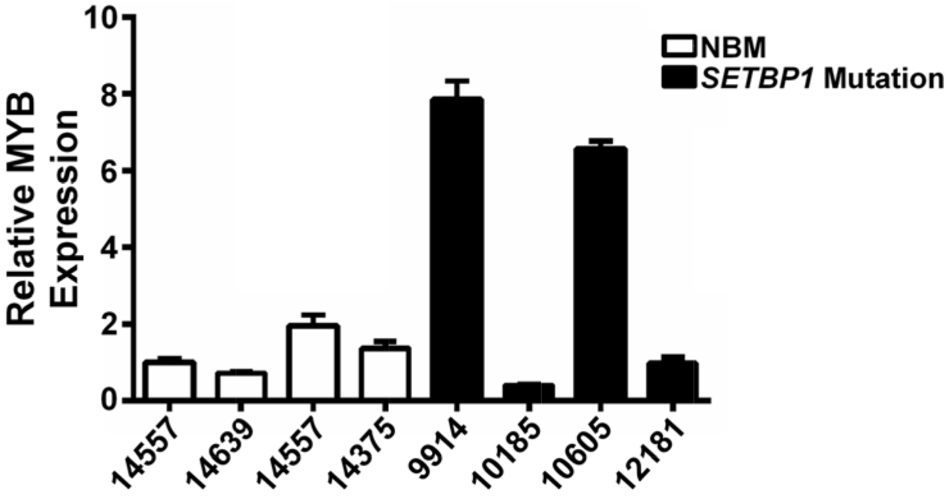

Supplementary Figure S10: mRNA expression of MYB in human myeloid malignancies with *SETBP1* missense mutations. Real-time PCR analysis of MYB mRNA levels in normal bone marrow samples (14557, 14639, 14557, and 14375) and bone marrow of different myeloid malignancy cases with *SETBP1* mutations including 9914 (secondary AML, p.D868Y), 10185 (RAEB-1, p.D868N), 10605 (RAEB-2, p.D868N), and 12181 (CMML, p.D868N). Relative expression levels were calculated by normalizing to GAPDH mRNA levels in the same sample and also in normal bone marrow sample 14557. The mean and SD of each relative expression level is shown.

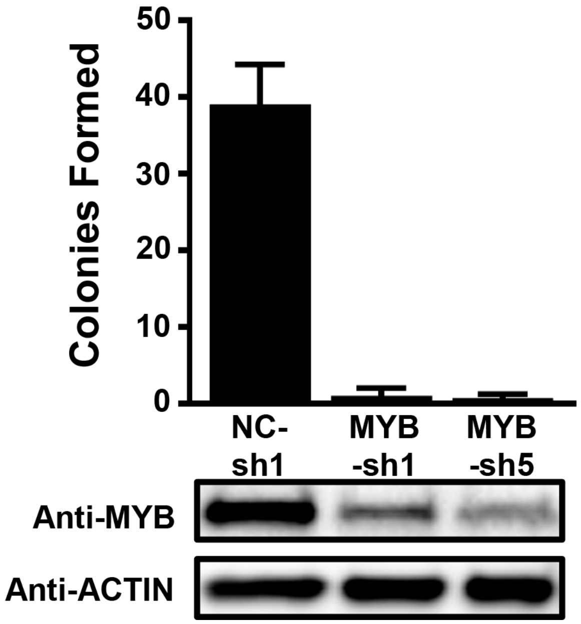

Supplementary Figure S11: Knockdown of *MYB* dramatically inhibits colony-forming potential of leukemia cells from patient 12181. Upper panel, mean and SD of colony numbers formed by 1×10^4 leukemia cells of patient 12181 at 48 hrs after infection with pLKO.1 lentiviral shRNAs targeting *MYB* (MYB-sh1 and MYB-sh5) or control shRNA (NC-sh1). Lower panels, Western blotting analysis of MYB and β-ACTIN protein levels in the same cells of the upper panel at 72 hrs after transduction. Before knockdown using lentiviral shRNAs, patient cells were expanded for 2 days on a layer of irradiated HS27 cells in DMEM with 15% FBS, 50 uM β-mercaptoethanol, 1% penicillin-streptomycin, and cytokines including 100ng/ml SCF, 10 ng/ml IL-3, 20ng/ml IL-6, 10 ng/ml TPO, and 10 ng/ml FLT3L. Puromycin (2 ug/ml) was added at 24 hours after transduction to select for transduced cells. Colony formation assays were performed on IMDM methylcellulose medium supplemented with same serum and cytokines. Colony numbers were counted after 7 days.

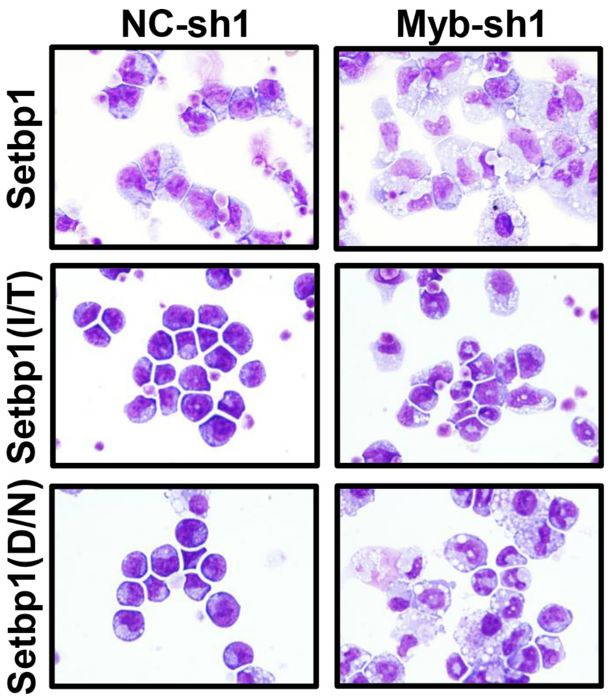

Supplementary Figure S12: Knockdown of *Myb* induces differentiation of *Setbp1* activation induced AML cells. Representative cytospins of *Setbp1*, *Setbp1 (I/T)*, and *Setbp1 (D/N)* induced mouse AML cells at 72 hrs after infection with lentiviral shRNA targeting *Myb* (Myb-sh1) or control shRNA (NC-sh1).

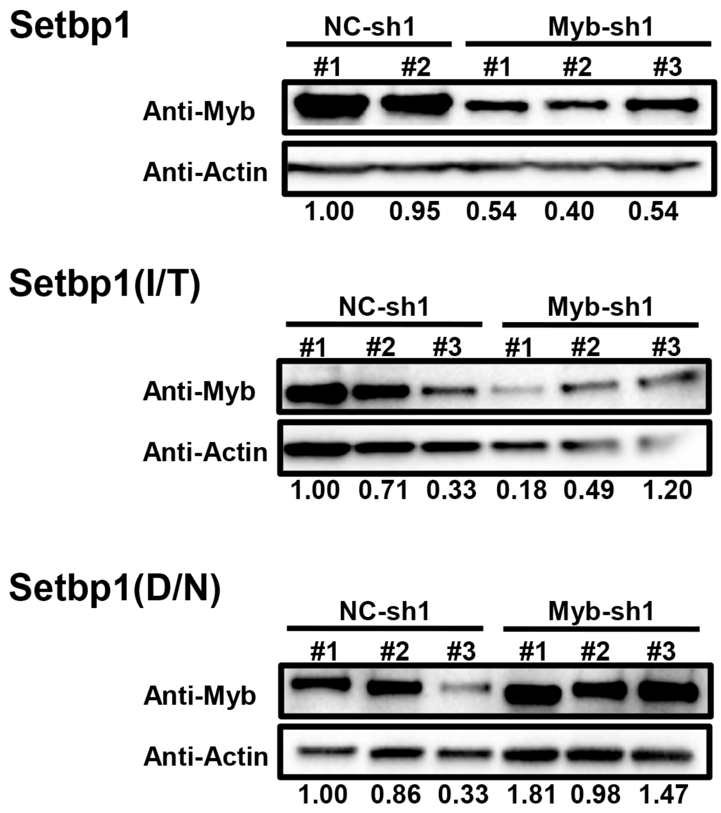

Supplementary Figure S13: Significant levels of Myb were detected in leukemias developed from *Myb* knockdown cells. Western blotting analysis of Myb and β-Actin protein levels in leukemic spleens of mice receiving leukemia cells induced by *Setbp1*, *Setbp1(I/T)*, or *Setbp1(D/N)* after transduction with negative control lentiviral shRNA (NC-sh1) or *Myb*-specific shRNA (Myb-sh1). Relative Myb protein levels in each sample after normalization to β-Actin levels are indicated. Protein bands were quantified using Quantity One data analysis software (Bio-Rad).

REFERENCES

1. Vishwakarma BA, Nguyen N, Makishima H, Hosono N, Gudmundsson KO, Negi V, Oakley K, Han Y, Przychodzen B, Maciejewski JP, Du Y. Runx1 repression by histone deacetylation is critical for Setbp1-induced mouse myeloid leukemia development. Leukemia. 2015. doi: 10.1038/leu.2015.200.

2. Oakley K, Han Y, Vishwakarma BA, Chu S, Bhatia R, Gudmundsson KO, Keller J, Chen X, Vasko V, Jenkins NA, Copeland NG, Du Y. Setbp1 promotes the self-renewal of murine myeloid progenitors via activation of Hoxa9 and Hoxa10. Blood. 2012; 119:6099–108. doi: 10.1182/blood-2011-10-388710.
